# Semantic Segmentation of the Malignant Breast Imaging Reporting and Data System Lexicon on Breast Ultrasound Images by Using DeepLab v3+

**DOI:** 10.3390/s22145352

**Published:** 2022-07-18

**Authors:** Wei-Chung Shia, Fang-Rong Hsu, Seng-Tong Dai, Shih-Lin Guo, Dar-Ren Chen

**Affiliations:** 1 Molecular Medicine Laboratory, Department of Research, Changhua Christian Hospital, Changhua 500, Taiwan; 2 Department of Information Engineering and Computer Science, Feng Chia University, Taichung 407, Taiwan; frhsu@fcu.edu.tw (F.-R.H.); torng1996@gmail.com (S.-T.D.); 3 Comprehensive Breast Cancer Center, Changhua Christian Hospital, Changhua 500, Taiwan; 109300@cch.org.tw; 4 School of Medicine, Chung Shan Medical University, Taichung 402, Taiwan

**Keywords:** breast cancer, deep convolutional neural network, semantic segmentation, ultrasonic imaging, computer-aided diagnosis

## Abstract

In this study, an advanced semantic segmentation method and deep convolutional neural network was applied to identify the Breast Imaging Reporting and Data System (BI-RADS) lexicon for breast ultrasound images, thereby facilitating image interpretation and diagnosis by providing radiologists an objective second opinion. A total of 684 images (380 benign and 308 malignant tumours) from 343 patients (190 benign and 153 malignant breast tumour patients) were analysed in this study. Six malignancy-related standardised BI-RADS features were selected after analysis. The DeepLab v3+ architecture and four decode networks were used, and their semantic segmentation performance was evaluated and compared. Subsequently, DeepLab v3+ with the ResNet-50 decoder showed the best performance in semantic segmentation, with a mean accuracy and mean intersection over union (IU) of 44.04% and 34.92%, respectively. The weighted IU was 84.36%. For the diagnostic performance, the area under the curve was 83.32%. This study aimed to automate identification of the malignant BI-RADS lexicon on breast ultrasound images to facilitate diagnosis and improve its quality. The evaluation showed that DeepLab v3+ with the ResNet-50 decoder was suitable for solving this problem, offering a better balance of performance and computational resource usage than a fully connected network and other decoders.

## 1. Introduction

In the clinical diagnosis of breast cancer, ultrasound (US) imaging is an important and common examination method. However, the diagnostic accuracy of breast US is highly dependent on the experience and technical ability of the operator. In general, the classification of ultrasound images usually relies on subjective evaluations by physicians based on their experience. Differences in skill, knowledge, and understanding of breast US techniques among operators may lead to differences in diagnosis. Therefore, computer-aided diagnosis recently become an important research direction to assist radiologist with image interpretation [1].

The classification of and segmentation between benign and malignant tumours on the basis of breast US is an important research field. In this regard, several studies obtained good performances by using machine learning or deep learning strategies [2,3]. However, from a clinical point of view, in comparison with the identification of benign and malignant lesions, the more important information is the compatibility of these findings with the predefined features of the standardised terminology in the Breast Imaging Reporting and Data System (BI-RADS) [4] in ultrasound, and the reporting of findings for each region [5,6]. The BI-RADS provides a lexicon of standardised terms for describing features observed in breast mass and radiology assessments; this approach is demonstrably effective for distinguishing between benign and malignant masses [7]. It also provides a standardised approach for reporting malignant or benign image findings to physicians and radiologists, thereby improving clinical diagnosis.

The use of a standardised BI-RADS lexicon as the basis of semantic analysis of breast ultrasound images for segmentation may be a possible solution to this problem. Semantic segmentation [8] is an important method for the establishment of image understanding on the basis of predefined semantic criteria [9]. Semantic segmentation attempts to understand the meaning of the entire image by dividing the content and recognising the location of each target or feature at the pixel level and then evaluating the findings. With the rapid development of deep learning and the increasing application of various and deeper network architectures, several semantic segmentation algorithms based on deep convolutional neural networks have been proposed [8,10]. Deep learning can reveal hidden but significant features in images and provide powerful reference data for clinical assessments. It can also decrease the bias in diagnosis between observers in the same case.

According to recent studies on the application of semantic segmentation and deep learning to breast ultrasound (as shown in Table 1), most of the studies still focus on establishing segmentation for tumour regions, while semantic segmentation is used as a strategy to enhance its effectiveness. It is worth mentioning that it is a complicated step to classify benign and malignant breast ultrasound and complete the grading by utilizing BI-RADS and related lexicons. It not only considers the imaging characteristics of the tumour area itself, but also considers that all malignant-related BI-RADS lexicons require both consideration and evaluation. Therefore, semantic analysis purely for tumour regions is different from our goal of study. Some studies [11,12] make good use of the feature of the semantic segmentation model that can create multiple semantic segmentations at the same time, and perform different image segmentation for different breast tissues or image features. Although a recent study [11] proposed the use of deep learning for semantic segmentation to promote the development of such research, it was limited by the small number of image samples included in the study, and more advanced deep learning methods to improve the performance of segmentation and recognition is also needed.

In this study, we applied semantic segmentation to detect malignant features based on the BI-RADS malignant lexicon definitions for breast ultrasound images by utilising an advanced semantic network to improve the limitations of previous studies. This study aimed to combine semantic segmentation in breast ultrasound with deep learning to detect several malignant-related image features in the same image simultaneously. The prediction result was then visualised to help physicians distinguish the malignancy in breast ultrasound, eventually improving the quality of clinical diagnosis.

## 2. Materials and Methods

### 2.1. Data Acquisition

This retrospective, cross-sectional study was approved by the Institutional Review Board of Changhua Christian Hospital, Changhua, Taiwan (No. 181235). The requirement for informed consent was waived. All experimental methods were supervised by the ethics committee, and conducted in accordance with the relevant guidelines and the Declaration of Helsinki.

Inclusion criteria for the enrolled patients were age ranging from 35 to 75 years and initial diagnosis (benign or malignant) at the institute. We excluded patients who accepted surgical treatment of breast cancer (any type of mastectomy) or mammoplasty, and those without essential imaging or clinical data. The GE Voluson 700 system (GE Healthcare, Zipf, Austria) was utilised to observe and acquire breast US images. For each image acquisition, two different angles of scan planes (usually in vertical) were obtained for each participant. The medical records of all enrolled patients, including the treatment, histological, and radiological findings, were also collected. Determination of benign or malignant disease was pathologically proven through fine-needle cytology, core-needle biopsy, or open biopsy.

**Figure 1:**
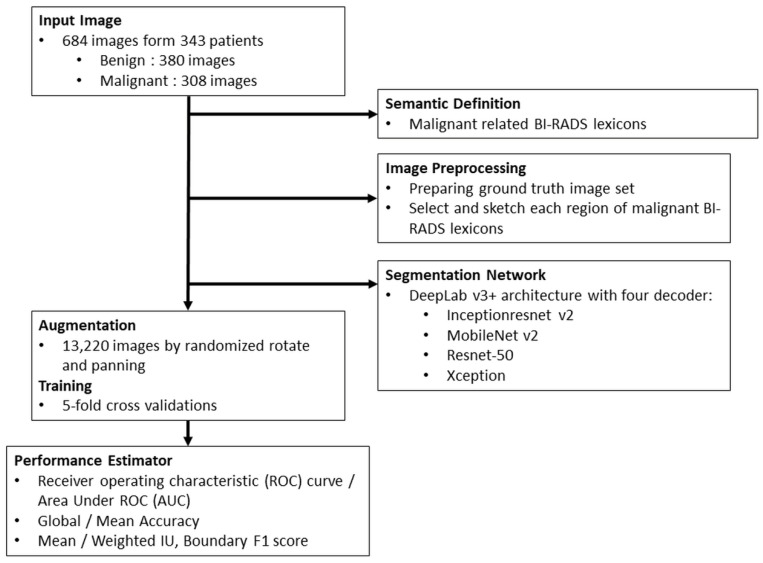
Flowchart of the study.

All identified solid masses in US images were described and categorised by standardised terms according to the category criteria of American College of Radiology (ACR) BI-RADS 5th Edition [18,19], and these descriptions were all verified by surgeons with over ten years of clinical experience. A flowchart of the data process, analysis, and performance estimation in this study is shown in Figure 1.

### 2.2. Definition of Semantics and Lexicons

The definition of semantics was based on the standardised BI-RADS lexicon for US provided by ACR. In the present study, this definition was derived from the high-frequency malignant lexicon corresponding to BI-RADS categories 4, 5, and 6. The six common and high-frequency malignant BI-RADS features included shadowing (posterior acoustic shadowing), taller-than-wide (the long axis is not parallel to the skin line), angular margins (circumscribed or indistinct tumour margin), micro-lobulation (masses have small undulations, like petals on a flower), hypoechogenicity (inhomogenicity of internal echo pattern, ill-defined or speculated margins), and duct extension (intraductal growth of breast cancer in single large duct extending toward nipple).

### 2.3. Image Pre-Processing

The input images did not contain a pre-selected tumour region or mark. In the pre-processing procedure, all unnecessary and irrelevant information was removed from the image, including the manufacturer marks, direction indicators, and text fields. Then, an experienced radiologist selected the malignant BI-RADS features for each region on the breast US image, and then filled them in different colours. Finally, the image was saved as the ground truth image and used for model training. The source of malignant features in each US image was based on the radiology report, and the correctness of the ground truth region and location was also confirmed by an experienced radiologist.

### 2.4. Semantic Segmentation Networks

**Figure 2:**
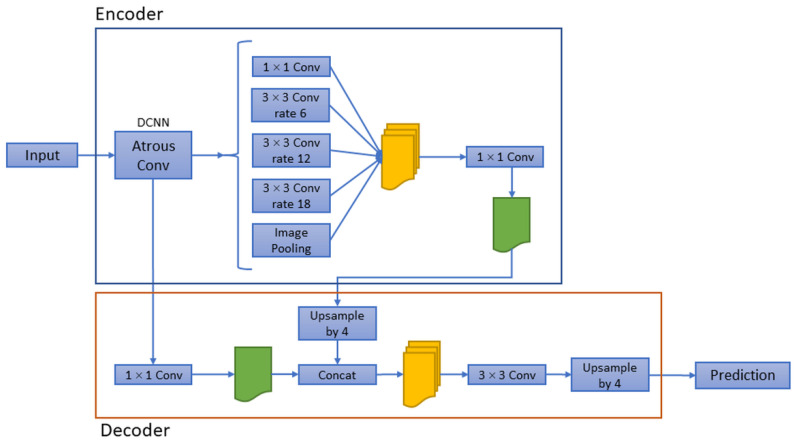
Network architecture of DeepLab V3+. The different rate (atrous rates) of convolutional layers in atrous convolution helps to enhance the field-of-view of the model.

DeepLab v3+ [20] architecture was utilised for the semantic segmentation network in this study. The most significant feature of the DeepLab architecture in comparison with other networks is the special ‘atrous’ algorithm and the fully connected conditional random field (CRF) [21], which improves the ability to accurately determine images for semantic segmentation. The ‘atrous’ algorithm mainly encodes multi-scale contextual information and enhances the ability to globally sample images and obtain features by applying atrous convolution, and the use of the CRF can accelerate the inference speed on the deep convolutional network. Through inserting equidistant holes between filter weights of the feature map, atrous convolution can extract denser feature maps from a pre-trained model. It also can increase the field-of-view of the model through the utilization of the larger ‘atrous rates’ (insert more holes between filter weights). The increase of the field-of-view of the model helps to enhance the ability to identify and classify individual features during semantic segmentation, which is a feature that was not available in similar semantic segmentation networks in the past. The DeepLab v3+ network is the latest generation of improvements in the DeepLab network family. The difference from the original architecture is the cancellation of the CRF mechanism and the use of the encoder-decoder architecture, which can expand and refine image segmentation by easily adding decoder modules. The network architecture of DeepLab v3+ is shown in Figure 2.

**Figure 3:**
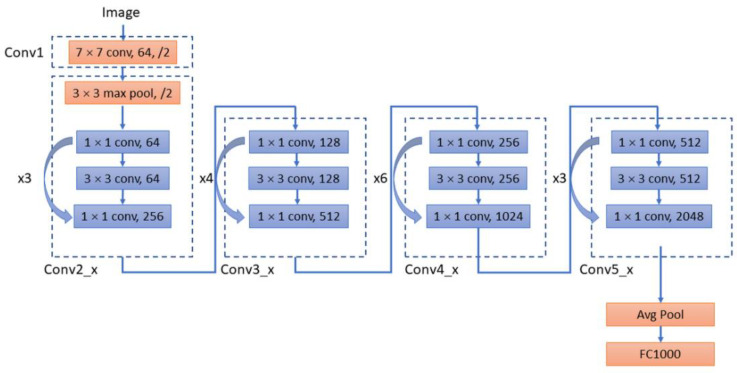
Network architecture of ResNet-50.

**Figure 4:**
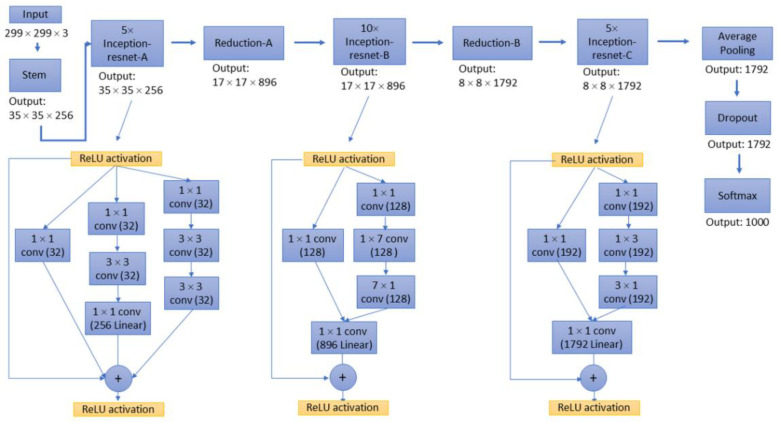
Network architecture of Inception-ResNet-v2. Illustrations of the reduction block structure were omitted.

**Figure 5:**
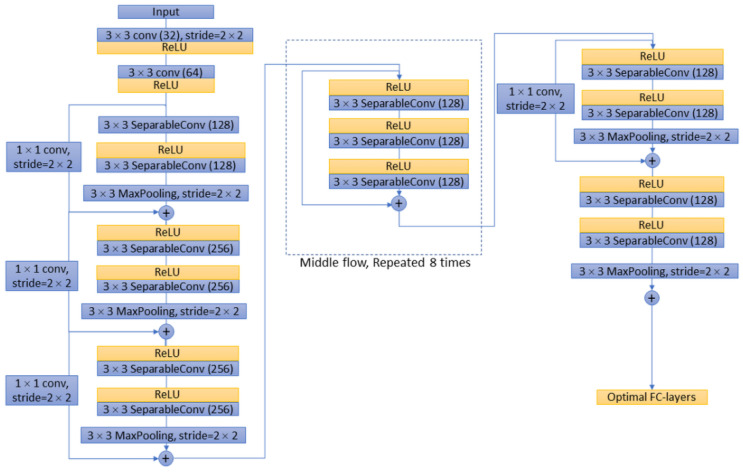
Network architecture of Xception.

**Figure 6:**
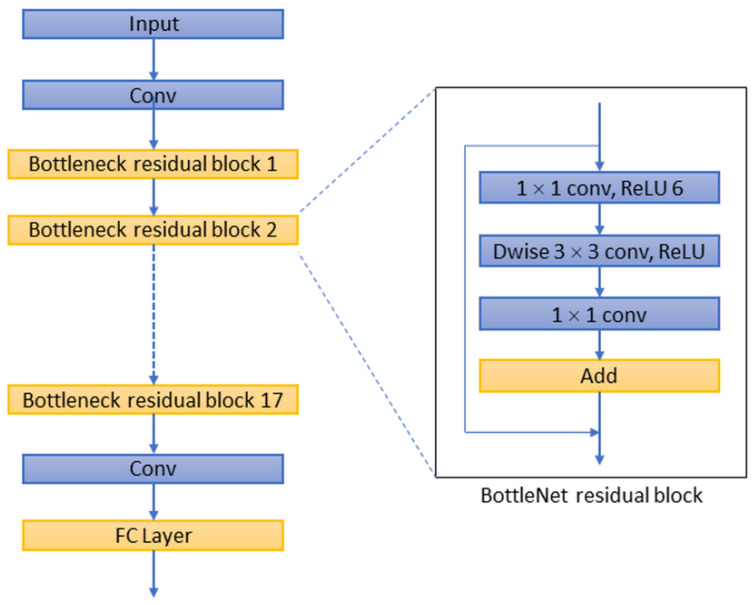
Network architecture of MobileNet-V2.

For the decoder network, four networks were selected and their performances were evaluated, including ResNet-50 [22], MobileNet-v2 [23], Inception-ResNet-v2 [24], and Xception [25]. As decoders, the implementation details of these deep convolutional networks are different from each other. For example, ResNet integrates image features in a highly stacked deep convolutional neural network through a residual network to avoid the problem of vanishing/exploding gradients (Figure 3). The Inception network uses ‘separable convolutions’, which is not based on the stacking of convolutional layers but instead builds a convolutional layer that can simultaneously learn the spatial dimensions and map cross-channel correlation and spatial correlation. Inception-ResNet is a variant of the Inception network architecture, adding the residual network characteristics of ResNet to the deep convolutional architecture of the traditional Inception network (Figure 4). Xception utilises depth-wise separable convolution to learn cross-channel correlations and spatial correlations and to generate features (Figure 5). The features of MobileNet include utilised inverted residuals and linear bottlenecks to build a convolution stack, and a filter feature as the source for further analysis, such as object detection or semantic segmentation (Figure 6). These different decoder networks showed different detection capabilities for image targets, and also influenced the correctness of image segmentation. The present study evaluated all of these decoder networks.

### 2.5. Training Protocol

The initial learning rate was set as 0.001, the learn rate schedule was set to ‘piecewise’, the drop period was set as 2, and the drop factor was set as 0.1. This reduces the learning rate by a factor of 0.1 every 2 epochs. The optimiser utilised the stochastic gradient descent with momentum [26]. No batch normalisation was performed. Image augmentation was applied to the dataset for improving the adaptability of the model. The utilised argumentation method includes random zooming (from 0.8× to 1.2×), rotation (from −90° to 90°), cropping, vertical/ horizontal flipping, and performing elastic distortion. Image crop size was 360 × 360 pixels. The other parameters of training included a batch size of 6 and 10 epochs. Each network model was trained by 5-fold cross-validation [27].

### 2.6. Performance Evaluation

The performance of semantic segmentation was estimated according to the overlap with the ground truth image dataset. The metrics utilised in estimation were based on those used in previous studies, and included the global accuracy, mean accuracy, mean/frequency-weighted intersection over union (IU) [28], and mean boundary F1 score (BF score) [29]. In brief, global accuracy is the ratio of correctly classified pixels to the total number of pixels, regardless of class. Mean accuracy indicates the percentage of correctly identified pixels for each class. The IU presents the ratio of correctly classified pixels to the total number of ground truth and predicted pixels in that class. The BF score indicates how well the predicted boundary of each class aligns with the true boundary. The semantic segmentation performance and accuracy of each network was estimated on the basis of the frequency-weighted IU in this study.

The diagnostic performance was also evaluated by plotting the receiver operating characteristic (ROC) curve and calculating the area under the ROC curve (AUC) [30]. A correct diagnosis was defined as >75% overlap of the ground truth and segmentation region.

### 2.7. Training Infrastructure

The computational platform used in this study employed an Intel Core i5-11400F processor (2.6 GHz hexa-core with up to 4.4 GHz Turbo Boost and 12 MB cache), 16 GB DDR4 RAM, and an NVIDIA RTX 3060 graphics card (12 GB video RAM). The NVIDIA Compute Unified Device Architecture (CUDA), version 11.2, and the NVIDIA CUDA Deep Neural Network library, version 8.2.0.53, were installed and utilised to enabled the graphics processing unit accelerated computation environment. All related programmes and metrics computing were implemented by utilising MATLAB 2021b update 5 with Image Processing, Computer Vision, and Deep Learning Toolbox (The Math Works, Natick, MA, USA).

## 3. Results

### 3.1. Characteristics of the Image Set

After the application of the exclusion criteria, the image dataset of this study included 684 images from 343 patients (380 benign and 308 malignant tumour images from 190 benign breast tumour patients and 153 malignant breast tumour patients) [11]. After the image-augmentation procedure, the total number of images increased to 13,220. For benign tumours, the three most common tissue types of solid nodes were fibroadenomas (55/190, 28.95%), fibrocystic changes (48/190, 25.26%), and fibroepithelial lesions (49/190, 25.79%). Among malignant tissue types, invasive ductal carcinoma (IDC) was the most common (119/153, 77.78%) histologic subtype, while the incidence of ductal carcinoma in situ (DCIS) was 22.22% (34/153). Detailed patient characteristics of the image dataset are shown in Table 2. The most common malignant BI-RADS features for the image dataset were angular margin (653/684, 95.47%) and taller-than-wide (102/684, 14.91%).

### 3.2. Semantic Segmentation Performance of Each Decoder

**Figure 7:**
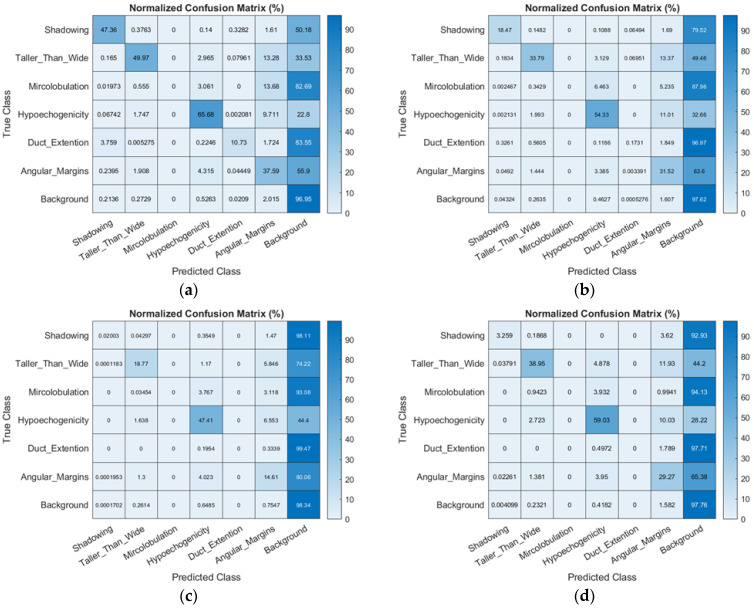
Normalised confusion matrix of the classification performance in DeepLab v3+ with the selected three decoders and FCN-32s, based on the selected six BI-RADS lexicons. The rate of correct recognition for each lexicon is shown in percentage. (**a**) Classification performance of DeepLab v3+ with ResNet-50; (**b**) classification performance of DeepLab v3+ with Inception-ResNet-v2; (**c**) classification performance of DeepLab v3+ with MobileNet-v2; (**d**) classification performance of the FCN-32s.

Table 3 presents the semantic segmentation performance and average run time of the four decoders in DeepLab v3+ that were evaluated in this study. For DeepLab v3+ with ResNet-50, the global accuracy was 90.67%, mean accuracy was 44.04%, weighted IU was 84.36%, and mean BF score was 59.79. The normalised confusion matrix of the classification performance of DeepLab v3+ with ResNet 50/Inception-ResNet v2/MobileNet v2 is also shown in Figure 7. The results indicate that DeepLab v3+ with ResNet 50 showed a better classification performance than other decoder networks, especially in identifying shadowing, taller-than-wide, and angular margin features. For the shadowing feature identification, the performance of DeepLab v3+ with ResNet 50 was 47.36% and better than those of DeepLab v3+ with Inception-ResNet v2 (18.47%) and MobileNet v2 (2.03%). For the taller-than-wide feature identification, the performance of DeepLab v3+ with ResNet 50 was 49.97%, which was better than those of DeepLab v3+ with Inception-ResNet v2 (33.79%) and MobileNet v2 (18.77%).

**Figure 8:**
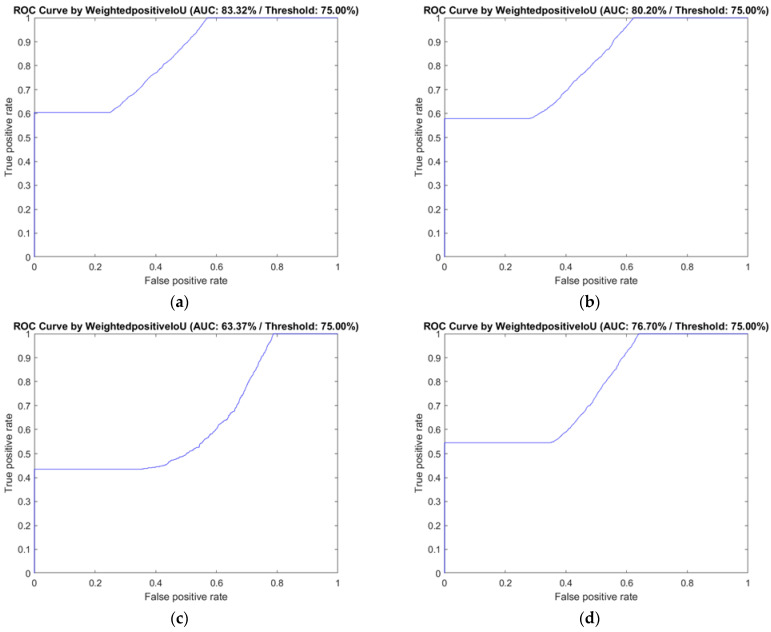
The ROC curve and AUC of the classification performance of DeepLab v3+ with the three selected decoders and FCN-32s, based on the six selected BI-RADS features. BI-RADS: Breast Imaging Reporting and Data System, ROC: receiver operating characteristic curve, AUC: area under curve. (**a**) Classification performance of DeepLab v3+ with ResNet-50; (**b**) classification performance of DeepLab v3+ with Inception-ResNet-v2; (**c)** classification performance of DeepLab v3+ with MobileNet-v2; (**d**) classification performance of FCN-32s.

The ROC curve and the AUC values of the four decoders are shown in Figure 8. In our evaluations, the AUC of DeepLab v3+ with Inception-ResNet v2 and ResNet 50 were all over than 80%, and the DeepLab v3+ with ResNet-50 showed the best performance (AUC = 83.32%). DeepLab v3+ with MobileNet v2 (AUC = 63.37%) and the Xception architecture (AUC = 59.54%) were not discussed further due to their low semantic segmentation performance.

## 4. Discussion

**Figure 9:**
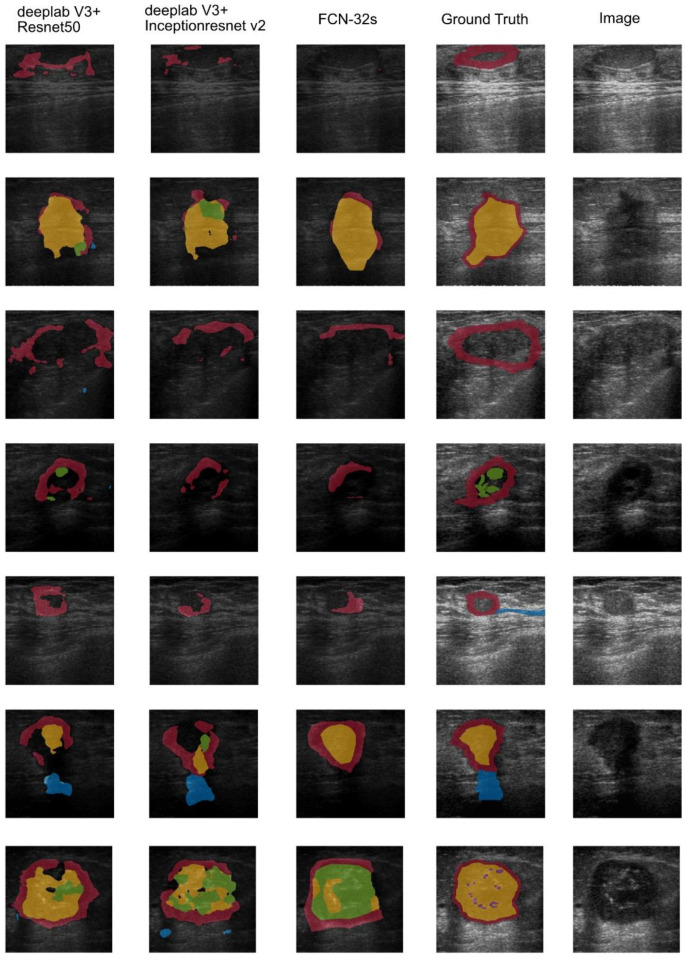
The sample of semantic segmentation visualisation for the malignant tumour ultrasound images after applying DeepLab v3+, and compared to FCN-32s. The sample US image and corresponding ground truth are shown in the two columns on the far right. The visualisation result from the first column on the left to the right: DeepLab v3+ with ResNet-50, DeepLab v3+ with Inception-ResNet-v2, FCN-32s. The semantically segmented regions based on BI-RADS lexicons are filled in the image with different colours. Red: angular margins, Green: hypoechogenicity, Yellow: taller-than-wide, Blue: duct extension, Navy Blue: shadowing, Purple: micro-lobulation.

A previous study [11] using a fully connected network (FCN) [31] indicated that this approach was a possible solution for BI-RADS lexicon detection; its global accuracy, weighted IU, and AUC were all over 85%. Therefore, an evaluation of the FCN-32s network was performed with the same image set and criteria. Figure 9 presents the visualisation result for each semantic segmentation network evaluated in this study. Each detected region of the malignant BI-RADS features in breast US images was filled with a different colour, allowing easy comparison of the differences between the semantic segmentation results of each model with the ground truth. From the example, we can find that FCN-32s could not generate a correct segmented region from malignant BI-RADS features in specific scenarios, and it is the major disadvantage. In addition, in comparison with the segmentation results obtained by DeepLab V3+ with ResNet-50 or Inception-ResNet-v2, the malignant feature regions segmented by FCN-32s were more fragmented. The results also showed that DeepLab v3+ with ResNet-50 or Inception-ResNet-v2 had more reasonable segmentation results than FCN-32s. The AUC of FCN-32s was 76.7%, slightly inferior to the results obtained using DeepLab v3+ with Inception-ResNet-v2 and ResNet-50.

In addition to its performance in semantic segmentation, two other significant benefits of DeepLab v3+ with ResNet-50 over FCN-32s were related to the training resource and exclusion time. Table 2 also presents the average training time in each fold. DeepLab v3+ with ResNet-50 required 130.74 min, and FCN-32s required 163.13 min. In terms of video memory usage, FCN-32s exhausted more than 90% of the video memory of the graphics card of the computing platform during the training period when using the default parameters in this study (11.1 GB), while DeepLab v3+ with ResNet-50 required approximately 70% of the memory usage (7.8 GB) of the FCN-32s. Overall, ResNet-50 as the decoding network in DeepLab v3+ uses less computing resources, and shows faster training and computing speed than FCN.

Analysis of the semantic segmentation performance and related decoder network architecture showed that the residue learning network and fully connected network (such as ResNet-50, Inception-ResNet-v2, FCN-32s) offered benefits in BI-RADS lexicon recognition. Although Xception and MobileNet have demonstrated good classification performance on large datasets such as ImageNet and JFT, their performance was not good in the BI-RADS malignant lexicon recognition for breast ultrasound images in this study. Some distinguishing features, such as the bottlenet residual block in MobileNet or the separable convolutional layer in the Xception network, did not improve the segmentation or classification performance in this study. The reason for the higher performance of ResNet-50/Inception-ResNet-v2 as decoders in DeepLab v3+ and FCN-32s may be that these networks provide a deep enough network for convergence of the features. This also reveals the particularity and difficulty of this problem.

In terms of image data of the same nature and the problem it is trying to solve, the performance of FCN-32s in this study is inferior to that reported in a previous study [23] after excluding differences in execution parameters. This may be attributable to the increased complexity associated with an increase in the cases (images). The findings also show that when a well-trained image recognition deep learning model is applied to image data sets of different sizes or different characteristics, its classification or recognition performance may degrade to a certain extent. In the comparison of FCN-32s with a normalised confusion matrix between DeepLab v3+ with ResNet-50/Inception-ResNet-v2, the recognition for the shadowing feature showed the most significant improvement. In FCN-32s, the accuracy (true/predicted class) was 3.26%, and the accuracy in DeepLab v3+ with ResNet-50 and Inception-ResNet-v2 was 47.36% and 16.47%. The recognition ability for the taller-than-wide feature and angular margin feature in DeepLab v3+ with ResNet-50/Inception-ResNet-v2 was also slightly better than those with FCN-32s.

The limitations of this study include the fact that more data (more than 1000 patients) are required to verify the availability of the trained model, and the features recognised in some specific BI-RADS lexicons were incomplete. As was the problem with reproducing the semantic segmentation performance of the FCN-32s in this study, a dataset with more real patient images would be useful to test the real performance for actual semantic segmentation. The image feature of malignant BI-RADS lexicon can be identified by a pre-trained model basically, but for some specific malignant features, the result of the segmented region is fragmented in comparison with the ground truth. This may cause some difficulties for radiologists in the grading of BI-RADS classification, so the results of this study still need to be improved. This issue may be resolved by developing more advanced model architectures or modifying the existing network architectures to address it. We will continue to seek breakthroughs in the future to address these limitations.

## 5. Conclusions

The incidence of breast cancer continues to remain high today. With the assistance of modern artificial intelligence technology and deep learning methods, giving radiologists a neutral and objective reference opinion and assisting them to accurately determine the lesions will help to promote good clinical practice. In this study, we achieved better semantic segmentation performance than that obtained in previous studies and architectures by using DeepLab v3+ with ResNet-50, with improvements in the recognition of various malignant features that are synonymous with the BI-RADS malignant terminology from US images. This also improves the consistency of image analysis results with clinical meaning. By training the single model for the recognition of multiple malignant BI-RADS lexicons, we can simultaneously identify, segment, and colorize all malignant feature regions detected on a breast ultrasound image, making their location and size clear at a glance. We believe that through the continuous development of such research, it will help to improve the efficiency and accuracy of diagnosis of radiologists in the future, and reduce their daily workload.

## Figures and Tables

**Table 1:** Summary of related semantic segmentation studies with their modalities and results.

References	Topic	Classes Identified	Dataset Size	Results
[11]	Incorporating the Breast Imaging Reporting and Data System lexicon with a fully convolutional network for malignancy detection on breast ultrasound	Malignant BI-RADS lexicons (shadowing, taller-than-wide, angular margins, micro-lobulation, hypo-echogenicity and duct extension)	378 (204 benign and 174 malignant images)	(In FCN-32s) Global Accuracy: 91.49 Mean IU: 32.82 Weighted IU: 85.35
[12]	Fuzzy semantic segmentation of breast ultrasound image with breast anatomy constraints	Fat layer, mammary layer, muscle layer and tumour region	325 (Mixed from two heterogeneity datasets)	(In FCN with fuzzy layer and proposed CRFs) Mean IU: 80.47
[13]	A comparative study of pre-trained convolutional neural networks for semantic segmentation of breast tumours in ultrasound	Benign/Malignant Tumour	3061 (Mixed from four heterogeneity datasets)	(In ResNet 18) F1 Score: 0.905 IU: 0.827
[14]	Semantic segmentation with DenseNets for breast tumour detection	Tumour Region	100 (From 78 patients)	Accuracy: 99.2 Dice coefficient: 0.83
[15]	Dilated semantic segmentation for breast ultrasonic lesion detection using parallel feature fusion	Tumour Region	780 * (Benign: 487, Malignant: 210, Normal: 133)	(In DenseNet-201) Accuracy: 98.97 Sensitivity: 100 Specificity: 98.63
[16]	Automatic semantic segmentation of breast tumours in ultrasound images based on combining fuzzy logic and deep learning—a feasibility study	Tumour Region	400 * (Benign: 200, Malignant: 200)	(In DeepLab V3+ with ResNet18) Global Accuracy: 96.79 Mean IU: 85.48 Mean BF Score: 74.14
[17]	Segmentation and recognition of breast ultrasound images based on an expanded U-Net	Tumour Region	192 (177 benign tumour images, 23 malignant tumour images)	Dice coefficient: 90.5 IU: 82.7

IU: intersection over union, BF score: boundary F1 score. * Same image data source.

**Table 2:** Patient and image characteristics.

Characteristics	Benign (n = 190)	Malignant (n = 153)
Age of patients (y)	47.35 (45.21–49.49)	53.51 (51.13–55.69)
Malignant tissues		
DCIS	-	34 (22.22%)
IDC	-	119 (77.78%)
Benign tumours		
LCIS	7 (3.68%)	-
Fibroadenoma	55 (28.95%)	-
Fibrocystic change	48 (25.26%)	-
Adenosis	5 (2.63%)	-
Fibroepithelial lesion	49 (25.79%)	-
Other	26 (13.69%)	-

DCIS: ductal carcinoma in situ; LCIS: lobular carcinoma in situ; IDC: invasive ductal carcinoma.

**Table 3:** Semantic segmentation performance and average run time results for DeepLab v3+ with ResNet-50/Inception-ResNet-v2/Mobilenet-v2/Xception and FCN-32s.

Network	Global Accuracy (%)	Mean Accuracy (%)	Mean IU (%)	Weighted IU (%)	Mean BF Score (%)	Average Run Time (mins)
DeepLab v3+ with ResNet-50	90.67	44.04	34.92	84.36	59.79	130.74
DeepLab v3+ with Inception-ResNet-v2	89.96	34.12	28.56	83.01	58.94	183.55
DeepLab v3+ with MobileNet-v2	89.13	25.59	22.19	80.88	57.47	96.15
DeepLab v3+ with Xception	88.64	25.23	21.36	80.48	57.26	141.52
FCN-32s	89.95	30.69	26.67	82.55	60.48	163.13

IU: intersection over union, BF score: boundary F1 score.

## Data Availability

The datasets generated during and analysed during the current study are not publicly available due to IRB and institutional restrictions, but are available from the corresponding author on reasonable request.
